# Application of Sofia Plus catheter tip shaping in the treatment of acute middle cerebral artery occlusion: A case control study

**DOI:** 10.1097/MD.0000000000035864

**Published:** 2023-11-10

**Authors:** Ning Han, Liang Ma, Yanzhao Xie, Guodong Xu, Yangjuan Jia, Ning Zhang, Hebo Wang

**Affiliations:** a Neurointerventional department, Hebei General Hospital, Shijiazhuang, Hebei, China; b Hebei Provincial Key Laboratory of Cerebral Networks and Cognitive Disorders, Shijiazhuang, Hebei, China; c Department of Emergency Medicine, Hebei General Hospital, Shijiazhuang, Hebei, China; d Department of Ultrasound, Hebei General Hospital, Shijiazhuang, Hebei, China; e Neurology Department, Hebei General Hospital, Shijiazhuang, Hebei, China.

**Keywords:** anterior circulation stroke, direct aspiration, ischemic stroke, thrombectomy

## Abstract

To explore the safety and efficacy of Sofia Plus distal access catheter tip shaping for treatment of acute middle cerebral artery embolism. This single-center retrospective study involved patients eligible for acute embolic middle cerebral artery occlusion from January 2020 to October 2021. They were divided into a shaping and non-shaping group according to whether the Sofia Plus catheter tip was shaped intraoperatively. Baseline data, preoperative Alberta Stroke Program Early Computed Tomography (ASPECT) score, National Institutes of Health Stroke Scale (NIHSS) score, onset-to-admission time, admission-to-puncture time, Sofia Plus-clot time, puncture-to-reperfusion time, surgical approach, and use of a stent for rescue thrombectomy were compared between the 2 groups. Postoperative symptomatic intracerebral hemorrhage and the modified Rankin scale score at the 90-day follow-up were observed. In total, 54 patients were enrolled in this study (shaping group, 26 patients; non-shaping group, 28 patients). Their mean age was 64.8 ± 14.6 years, and the proportion of men was 68.5% (37/54). Successful recanalization was achieved in all patients. There were no differences in the baseline data (age, sex, history, pre-admission ASPECT score, or NIHSS score) between the shaping and non-shaping groups. Patients treated with a shaped Sofia Plus catheter had a shorter Sofia Plus-clot time [median (25th, 75th percentile: 4 (4, 7) vs 10.5 (5.25, 14) min, *P* = .006] and puncture-to-reperfusion time [16.5 (12, 30.5) vs 26 (16.25, 38.25) min, *P* = .036]. There were significant differences in the surgical approaches between the 2 groups. The rates of a favorable outcome (57.7% vs 64.3%, *P* = .62) and postoperative symptomatic intracerebral hemorrhage (7.7% vs 3.6%, *P* = .60) were not significantly different between the groups. Sofia Plus catheter tip shaping improved catheter trafficability and reduced the operative time. It was safe and effective for treatment of acute middle cerebral artery thrombotic occlusion.

## 1. Introduction

Stroke is a leading cause of death in China, and the prevalence continues to increase.^[1]^ Among adults aged 40 years or older, the incidence and mortality rate of stroke in China in 2020 were 505.2 per 100,000 person-years, and 343.4 per 100,000 person-years, respectively.^[1]^ Cerebral vascular perfusion therapy timely can reduce mortality and disability rates. Several randomized controlled studies have demonstrated the efficacy of mechanical thrombectomy for acute anterior circulation large-vessel occlusion.^[2–7]^ Stent retriever thrombectomy is currently considered the first-line mechanical thrombectomy treatment. However, increasingly more evidence is suggesting that contact aspiration is not inferior to use of a stent retriever.^[8,9]^ A recent study showed that contact aspiration therapy was more effective for embolic large-vessel occlusion.^[10]^ In addition, the contact aspiration technique has the advantage of a short operative time and low operative cost.

The Sofia Plus catheter (MicroVention, Aliso Viejo, CA) can be used both as an intermediate catheter and as an aspiration catheter. Because of its weak supporting ability, it is less commonly used as an intermediate catheter in the clinical setting. Because of its good trafficability, however, it is widely used as an aspiration catheter. In some patients, the Sofia Plus aspiration catheter can directly approach a thrombus with a bare catheter because of its good trafficability. However, in most patients, it is necessary to use coaxial technology with a micro-guidewire and microcatheter to move the aspiration catheter close to the thrombus. No reports have indicated whether steam-shaping of the tip of the Sofia Plus catheter can improve its trafficability. This study was performed to investigate the safety and efficacy of Sofia Plus catheter tip shaping in the treatment of acute embolic middle cerebral artery occlusion.

## 2. Materials and methods

### 2.1. Patients

**Figure 1.**
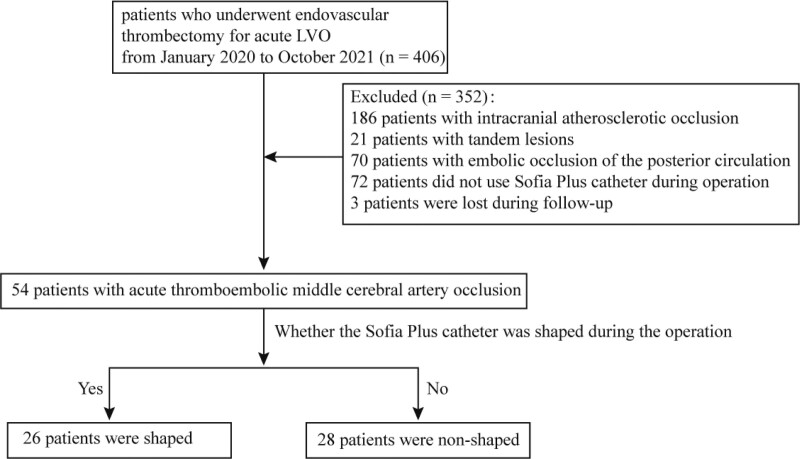
Flowchart of patient selection. LVO = large vessel occlusion.

From January 2020 to October 2021, 406 patients with acute ischemic stroke underwent endovascular treatment. Of these patients, 186 had intracranial large atherosclerotic occlusion, 21 had tandem lesions, 70 had embolic posterior circulation occlusion, and 72 were not treated with a Sofia Plus catheter during the operation. The remaining 57 patients met the trial inclusion criteria, and 3 of these patients were lost to follow-up (Fig. 1). They were divided into a shaping group and a non-shaping group according to whether the tip of the Sofia Plus catheter was shaped during the operation. The inclusion criteria were as follows: age of > 18 years, onset time of < 6 hours, diagnosis of middle cerebral artery occlusion caused by embolism, preoperative Alberta Stroke Program Early Computed Tomography (ASPECT) score of > 6, National Institutes of Health Stroke Scale (NIHSS) score before admission of > 6, thrombolytic therapy with simultaneous bridging endovascular therapy for patients eligible for intravenous thrombolysis, and preoperative vascular-related examinations (cranial magnetic resonance angiography, computed tomography angiography) to determine the presence of middle cerebral arterial occlusion. The exclusion criteria were as follows: active bleeding, middle cerebral artery occlusion caused by atherosclerosis and dissection (localized stenosis or contrast stasis found after intraoperative recanalization), and serious organic diseases such as heart, lung, or kidney disease. The local ethics committee approved this study.

### 2.2. Data collection and outcome evaluation

Clinical data were collected, including age, sex, history (hypertension, diabetes mellitus, coronary artery disease, hyperlipidemia, atrial fibrillation, prior stroke, and smoking), NIHSS score at admission, ASPECT score, onset-to-admission time, admission-to-puncture time, catheter thrombus time, puncture-to-reperfusion time, use of intravenous tissue plasminogen activator, mode of anesthesia, surgical approach used to advance the Sofia Plus catheter to the proximal end of the thrombus (bare catheter technology, microcatheter coaxial technology, or stent anchoring technology), and stent rescue mechanical thrombectomy (if the thrombus escaped to the M2 segment, a stent could be used for rescue thrombectomy). Angiographic modified Thrombolysis in Cerebral Infarction grade ≥ 2b was defined as successful recanalization. Symptomatic intracerebral hemorrhage within 7 days after the operation and the modified Rankin scale score at the 90-day follow-up were observed. A score of 0 to 2 was defined as a good outcome, and a score of 3 to 6 was defined as a poor outcome.

### 2.3. Treatment protocol

The Sofia Plus catheter was used to treat acute embolic middle cerebral artery occlusion. The surgeon decided whether to use the Sofia Plus catheter. The use of local or general anesthesia was determined based on the patient level consciousness and the surgeon preference. However, our principle was to use local anesthesia as much as possible because patients with embolization have poor collateral compensation and need to be recanalized as soon as possible.

**Figure 2.**
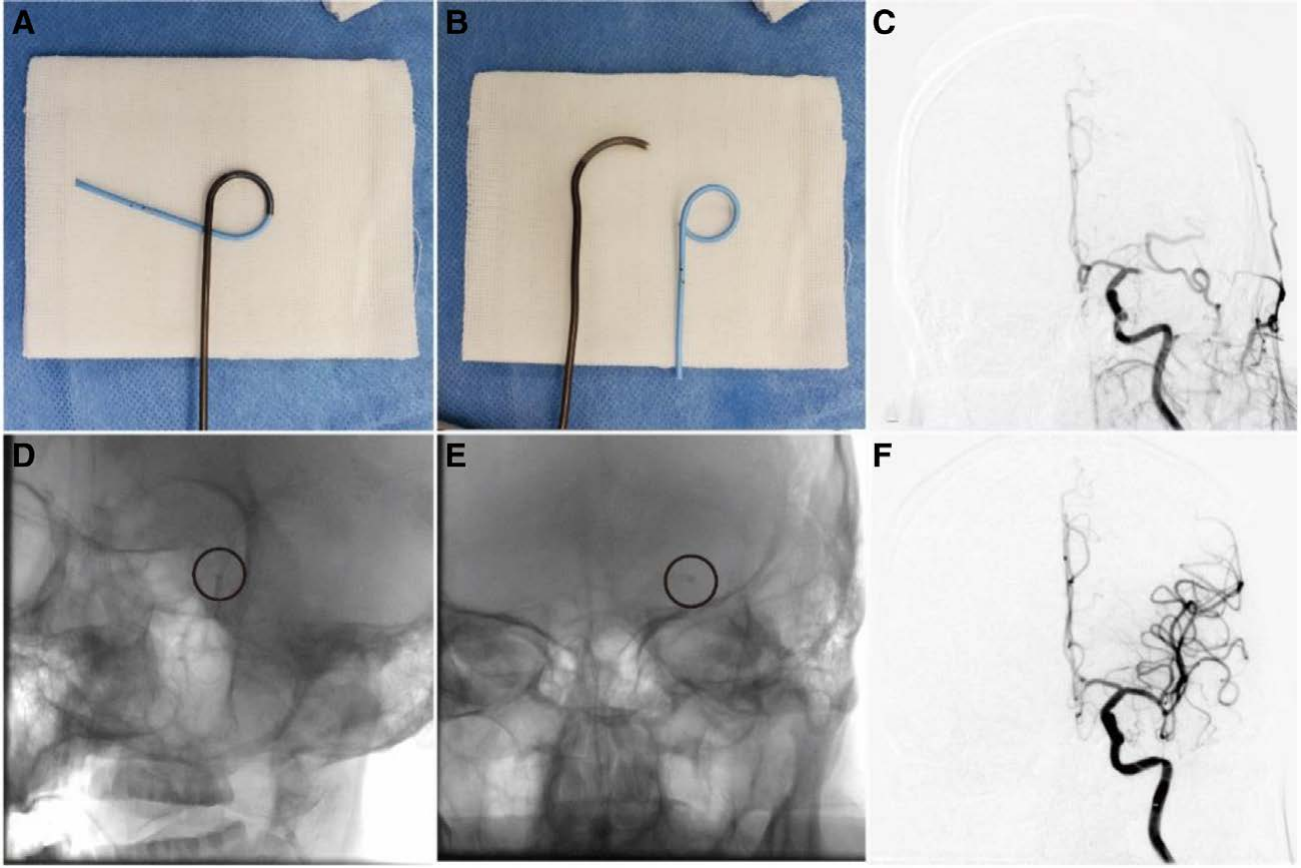
Representative patient with acute embolic occlusion of the left middle cerebral artery. (A, B) The Sofia Plus catheter tip was steam-shaped with a pigtail catheter. (C) Occlusion of the M1 segment of the left middle cerebral artery. (D) The shaped Sofia Plus catheter tip smoothly passed through the ophthalmic artery. (E) The Sofia Plus catheter tip reached the proximal occlusion. (F) Complete recanalization of the left middle cerebral artery was achieved.

**Figure 3.**
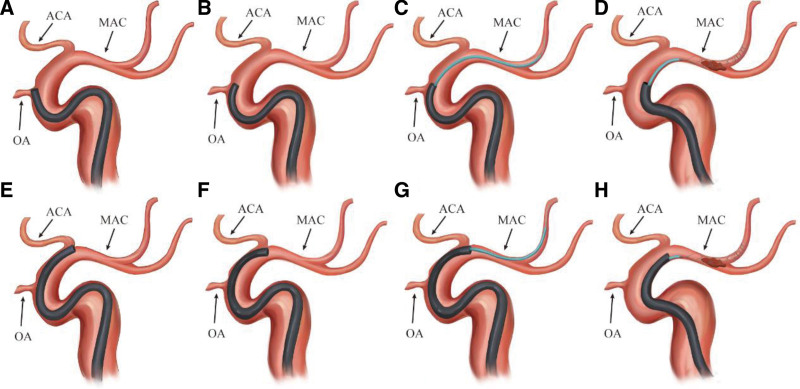
(A) The Sofia Plus catheter without tip shaping could not smoothly pass through the opening of the OA. (B). The Sofia Plus catheter with tip shaping smoothly passed through the opening of the OA. (C, D) Coaxial technology and stent anchoring technology enabled the Sofia Plus catheter to smoothly pass through the opening of the OA. (E) The Sofia Plus catheter without tip shaping could not smoothly pass through the opening of the ACA. (F) The Sofia Plus catheter with tip shaping smoothly passed through the opening of the ACA. (G, H) Coaxial technology and stent anchoring technology enabled the Sofia Plus catheter to smoothly pass through the opening of the ACA. ACA = anterior cerebral artery, OA = ophthalmic artery, MAC = middle cerebral artery.

We first placed the tip of a Neuron MAX long sheath (Penumbra Inc., Alameda, CA) in the C1 segment; the tip of the long sheath could then be pushed up further according to the patient vascular condition. Whether the tip of the Sofia Plus catheter was shaped was mainly determined by the operator preference. Our procedures were mainly performed by 2 neurointerventional specialists (M.L.,7 years’ experience; X.G.D., 12 years’ experience). Regardless of whether the Sofia Plus catheter tip was shaped, in all thrombectomy procedures we first attempted to use the bare catheter to reach the thrombus site. If shaping was required during the operation, a 5-Fr pigtail catheter could be used to insert the tip of the 6-Fr Sofia Plus catheter, and the degree of shaping could be selected according to the condition of the blood vessels (Fig. 2, Supplemental Video 1, http://links.lww.com/MD/K552). If the bare catheter was difficult to ascend, the proximal end of the occluded segment could not be reached smoothly. We tried to navigate the Sofia Plus catheter tip proximal to the face of the thrombus by the microcatheter coaxial technique. If the microcatheter coaxial technique still failed to reach the proximal thrombus, the stent anchoring technique was used. The most common reason for the inability to smoothly place the catheter tip was the “step effect” of the catheter, which was stuck at the opening of the ophthalmic artery or the anterior cerebral artery. The treatment strategies were steam-shaping of the catheter tip, coaxial technique, and stent anchoring technique (Fig. 3). After the tip of the Sofia Plus catheter reached the proximal face of the thrombus, a 20-mL syringe was used to perform negative-pressure aspiration. After the thrombus was withdrawn, angiography was performed to determine whether the thrombus had escaped. If the blood vessel was still not recanalized after repeating the above operation 3 times, a stent was used to remove the thrombus. If the M2 segment was occluded, the stent was also used for thrombectomy. If the thrombus escaped beyond the M2 segment, no further treatment was performed. Figure 1 shows a representative case involving a patient with acute embolic occlusion of the left middle cerebral artery.

### 2.4. Statistical analysis

The data were statistically analyzed using SPSS 26.0 software (IBM Corp., Armonk, NY). Count data are expressed as rate (%), and the chi-square test or Fisher exact probability method was used for comparison between the groups. Measurement data conforming to a normal distribution are expressed as mean ± standard deviation, and the independent-samples *t* test was used for comparison between groups. Measurement data not conforming to a normal distribution are expressed as median (25th, 75th percentile), and the non-parametric test was used for comparison between groups. A *P* value of < .05 was considered statistically significant.

## 3. Results

### 3.1. Baseline characteristics

In total, 54 patients were included in this study. Their mean age was 64.78 ± 14.62 years, median NIHSS score was 13.5 (11.75, 16), and median cranial computed tomography ASPECT score was 9 (8, 9). The patients were assigned to 2 groups according to whether the Sofia Plus catheter tip was shaped during the operation (26 patients in the Sofia catheter shaping group, 28 patients in the non-shaping group). There were no significant differences between the 2 groups in baseline data, such as age; sex; hypertension; diabetes mellitus; coronary artery disease; hyperlipidemia; atrial fibrillation; high-risk factors for cerebrovascular diseases, such as a history of stroke and smoking (*P* > .05); preoperative NIHSS score; ASPECT score; onset-to-admission time; and admission-to-puncture time (*P* > .05) (Table 1).

**Table 1:** Comparison of variables between Sofia Plus catheter shaping and non-shaping groups.

Variables	Overall (n = 54)	Shaping group (n = 26)	Non-shaping group (n = 28)	*P* value
Age (yr)	64.78 ± 14.62	67 ± 14.5	62.7 ± 14.7	.29
Male	37 (68.5%)	19 (73.1%)	18 (64.3%)	.49
Medical history				
Hypertension	28 (51.9%)	12 (46.2%)	16 (57.1%)	.42
Diabetes mellitus	7 (13%)	3 (11.5%)	4 (14.3%)	.76
Hyperlipidemia	23 (42.6%)	14 (53.8%)	9 (32.1%)	.11
Coronary artery disease	18 (33.3%)	8 (30.8%)	10 (35.7%)	.70
Atrial fibrillation	43 (79.6%)	21 (80.8%)	22 (78.6%)	.84
Smoking	20 (37%)	11 (42.3%)	9 (32.1%)	.44
prior stroke history	15 (27.8%)	6 (23.1%)	9 (32.1%)	.46
Preoperative ASPECT score	9 (8, 9)	8 (8, 9)	9 (8, 9)	.27
Preoperative NIHSS score	13.5 (11.75, 16)	14 (12, 19)	10 (13, 15)	.40
Use of IV tPA	14 (25.9%)	5 (19.2%)	9 (32.1%)	.28
Onset-to-admission time (min)	180 (120, 300)	195 (120, 255)	180 (120, 300)	.61
Admission-to-puncture time (min)	40.5 (34, 48)	42 (34, 50.5)	39 (34, 48)	.51

Data are presented as mean ± standard deviation, n (%), or median (25th, 75th percentile).

ASPECT = Alberta Stroke Program Early Computed Tomography, NIHSS = National Institutes of Health Stroke Scale, IV tPA = intravenous tissue plasminogen activator.

### 3.2. Procedural-related characteristics

Successful recanalization was achieved in all 54 patients without vessel-related complications (e.g., arterial dissection, perforation, or spasm). There was no significant difference in the type of anesthesia between the 2 groups (*P* > .05). The Sofia Plus-clot time was significantly different between the shaping group and the non-shaping group (4.0 vs 10.5 minutes, *P* = .006). The puncture-to-reperfusion time was also significantly different between the groups (16.0 vs 23.5 minutes, *P* = .039).

**Figure 4.**
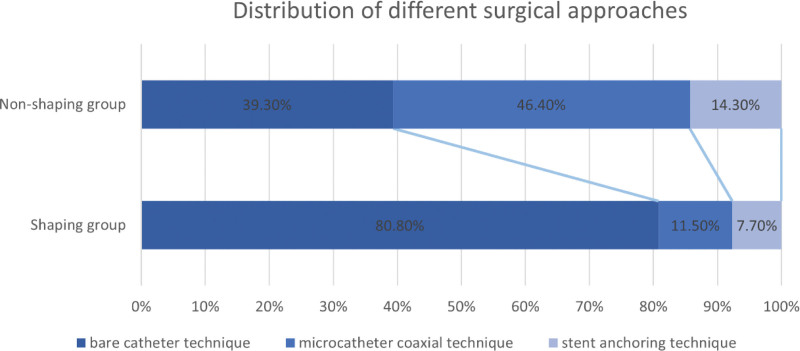
Distribution of different surgical approaches in the shaping group and non-shaping group.

During the intervention, the bare catheter technique was used significantly more often in the shaping than non-shaping group (80.8% vs 39.3%, *P* < .01). The distribution of surgical approaches in the shaping and non-shaping groups is shown in Figure 4. The use of rescue stent retrieval was not different between the groups (15.4% vs 21.4%, *P* = .57). Symptomatic intracerebral hemorrhage occurred less frequently in the non-shaping group; however, the difference was not significant (7.7% vs 3.6%, *P* = .60). Additionally, the incidence of a modified Rankin scale score of 0 to 2 at 90 days was not significantly different between the 2 groups (57.7% vs 64.3%, *P* = .62) (Table 2).

**Table 2:** Procedural and clinical outcomes of patients with and without Sofia Plus catheter shaping.

Variables	Overall (n = 54)	Shaping group (n = 26)	Non-shaping group (n = 28)	*P* value
Procedural dates				
Local anesthesia	34 (63%)	15 (57.7%)	19 (67.9%)	.44
Sofia Plus-clot time (min)	6 (4, 12)	4 (4, 7)	10.5 (5.25, 14)	.006
Puncture-to-reperfusion time (min)	24.5 (14.75, 34.25)	16 (12, 21.25)	23.5 (15.25, 26)	.039
Surgical approach				<.01
•Bare catheter technique	32 (59.3%)	21 (80.8%)	11 (39.3%)	
•Microcatheter coaxial technique	16 (29.6%)	3 (11.5%)	13 (46.4%)	
•Stent anchoring technique	6 (11.1%)	2 (7.7%)	4 (14.3%)	
Rescue stent retriever	10 (18.5%)	4 (15.4%)	6 (21.4%)	.57
Clinical Outcome				
Symptomatic intracerebral hemorrhage	3 (5.6%)	2 (7.7%)	1 (3.6%)	.60
mRS score of 0-2 at 90 d	33 (61.1%)	15 (57.7%)	18 (64.3%)	.62

Data are presented as n (%) or median (25th, 75th percentile).

mRS = modified Rankin scale.

## 4. Discussion

The contact aspiration technique in thrombectomy was first reported by Turk et al^[11]^ in 2014. The study showed that 75% (28/37) of patients could be recanalized by the aspiration technique alone and that the recanalization time was shorter than when using a stent retriever.^[11]^ An observational study on the contact aspiration technique and stent retriever showed that the contact aspiration technique was able to recanalize only 50.8% (63/124) of patients and that the contact aspiration technique combined with a rescue stent retriever improved the recanalization rates; however, this study did not specifically classify the types of occlusions.^[12]^ A recent study of contact aspiration techniques for embolic and intracranial atherosclerotic lesions showed that patients with embolic lesions had a higher chance of successful recanalization immediately after aspiration thrombectomy (64.71% vs 27.91%) and required less use of rescue therapy (35.29% vs 70.09%) than patients with intracranial atherosclerotic lesions.^[10]^ In the present study, we retrospectively analyzed the application of steam-shaping of the Sofia Plus catheter tip in the treatment of embolic middle cerebral artery occlusions.

The keys to the direct aspiration technique are the trafficability and diameter of the aspiration catheter. The trafficability of the Sofia Plus catheter is greatly enhanced by the unique braided design of its tip. The rate of successful access to the proximal end of the thrombus under the guidance of a micro-guidewire and a microcatheter has been reported to reach 92.7% (38/41) without catheter-related surgical complications such as vasospasm or dissection.^[13]^ In the present study, we first attempted to place the Sofia Plus catheter tip proximal to the thrombus using the bare catheter technique. The bare catheter successfully reached the proximal end of the thrombus in 63% (34/54) of patients, and the bare catheter was in place in 88.5% (23/26) of the patients in the shaping group and 39.3% (11/28) of those in the non-shaping group. No catheter-related complications were found in this study. This suggests that the shaped Sofia Plus catheter not only enhanced the trafficability of the catheter but also did not compromise the safety of the catheter.

Smith et al^[14]^ studied several parameters for successful implementation of the contact aspiration technique and found that strong suction force at the tip of the aspiration catheter was required to draw the thrombus into the catheter and that a larger diameter of the tip of the aspiration catheter provided greater suction force at the tip. The distal diameter of the Sofia Plus catheter can reach 0.070 inches, which can provide sufficient suction force. In the present study, 83.3% (45/54) of patients achieved complete recanalization by aspiration, and 10 patients underwent rescue thrombectomy using a stent. However, some studies showed that a larger diameter of the aspiration catheter was not associated with more effective thrombectomy. Medium-diameter aspiration catheters showed higher efficiency of thrombectomy, whereas large-diameter aspiration catheters were difficult to place, thereby increasing the time of thrombectomy.^[15]^

Despite the current improvements in the delivery of aspiration catheters, placing the tip of a large-diameter aspiration catheter to the proximal end of the thrombus remains challenging. This is especially true when the catheter passes through the origin of the ophthalmic artery and the A1 segment at a precarious angle, resulting in failure to smoothly pass the catheter in some patients (Fig. 3). Previous articles have reported that steam-shaping of the Sofia Plus catheter tip may improve its trafficability,^[16]^ but no relevant studies have been performed. This study showed that steam-shaping significantly improved the trafficability of the aspiration catheter. In the steam-shaping group, 80.8% (21/26) of catheters were successfully placed using only the bare catheter, while only 39.3% (11/28) were successfully placed in the non-shaping group. When placement of a bare catheter is difficult, we recommend a micro-guidewire combined with a microcatheter to place the aspiration catheter using the coaxial technique. When placement is particularly difficult, the stent anchoring technique can be used to place the aspiration catheter. The direct aspiration technique can reduce the operative time because it does not require the manipulation of a micro-guidewire or microcatheter, greatly reducing the operative time.

This study confirmed that shaping of the Sofia Plus catheter tip can improve its trafficability. However, there are some limitations to this study. First, only patients with middle cerebral artery occlusion caused by thromboembolism were enrolled; those with middle cerebral artery occlusion caused by intracranial atherosclerosis were not included. Additionally, if balloon dilation or stent placement is required, there may be insufficient proximal support and the balloon tip may not be in place because of the weak support and long length of the Sofia Plus catheter. Second, the sample size of this study was small, and the statistical results may be biased. Third, there may be errors in the angle of the shaping. We used the tip of the pigtail catheter tip for shaping; the angle after shaping may not be consistent, leading to possible bias in the results.

This study demonstrated that steam-shaping of the Sofia Plus catheter tip is safe and effective in patients with embolic middle cerebral artery occlusion and that shaping improves the trafficability of the intermediate catheter, thereby reducing the catheter placement time and relatively reducing the cost of surgery. In addition, other types of aspiration catheters may also be steam-shaped at the tip to improve the catheter trafficability if permitted by the manufacturer specifications.

## 5. Conclusion

Our study suggests that Sofia Plus catheter tip shaping can not only improve the surgical efficiency of acute embolic middle cerebral artery occlusion but also does not increase the surgical risk. However, a large-sample study is needed to determine its reliability.

## Acknowledgments

We thank Angela Morben, DVM, ELS, from Liwen Bianji (Edanz) (www.liwenbianji.cn) for editing the English text of a draft of this manuscript.

## Author contributions

**Conceptualization:** Ning Han.

**Data curation:** Ning Han, Liang Ma.

**Formal analysis:** Yanzhao Xie.

**Investigation:** Guodong Xu.

**Methodology:** Guodong Xu.

**Project administration:** Hebo Wang.

**Resources:** Hebo Wang.

**Software:** Yangjuan Jia.

**Supervision:** Ning Zhang.

**Validation:** Liang Ma, Guodong Xu.

**Writing – original draft:** Ning Han.

**Writing – review & editing:** Liang Ma.

## Supplementary Material


